# Use of a Trephine to Extract a Fractured Corail Femoral Stem During Revision Total Hip Arthroplasty: Tips From Our Case Report

**DOI:** 10.7759/cureus.52996

**Published:** 2024-01-26

**Authors:** Abhimanyu Singh, Srikanth Gandavaram, Kuntal Patel, Deepak Herlekar

**Affiliations:** 1 Orthopaedics and Trauma, Royal Lancaster Infirmary, Lancaster, GBR

**Keywords:** hip total arthroplasty, fracture, revision total hip arthroplasty, femoral stem, trephine

## Abstract

Despite the significant advancements in the field of total hip arthroplasty (THA) and prosthesis designs, fracture of the modular femoral stem after THA can occur rarely and needs attention. Orthopaedic surgeons face a daunting task when it comes to the removal of a broken stem. The use of a trephine reamer has been evaluated for extracting the distal femoral stem, and this case report tries to address some key tips for the same.

A 67-year-old obese male, without any major medical comorbidities, presented to the Royal Lancaster Infirmary orthopaedic outpatient department with a complaint of acute-on-chronic right anterior thigh pain that worsened over a few weeks. He had a history of bilateral staged uncemented THA done around 12 years ago. The plain radiological images confirmed the presence of a fracture of the Corail femoral stem. A posterior approach was used to dislocate the hip and the distal broken part of the stem was removed using trephines. Reamers were used and care was taken to prevent thermal necrosis by using intermittent saline lavage. After the removal of the fractured femoral stem, a cemented femoral revision THA was performed, which was uneventful. The patient walked without any aid or thigh pain postoperatively during his last follow-up.

Using trephines to remove broken femoral stems is an effective and safe method. Intraoperative measures can help in avoiding heat necrosis while using a trephine reamer for extracting the fractured femoral stem. Regular follow-up and counselling are important after THA, to avoid a delayed diagnosis of non-traumatic femoral stem fractures.

## Introduction

Orthopaedic surgeons worldwide commonly perform total hip arthroplasty (THA). An occurrence of a fracture in the modular femoral stem after THA, without any associated trauma, is rare, and seldom evaluated in scientific literature. This is particularly noteworthy considering the significant advancements in prosthesis designs that have taken place in recent times. However, the occurrence of a femoral stem fracture in THA patients continues to be a significant issue. Surgeons face a difficult task when trying to remove a broken part, particularly when there is a free proximal part and a securely attached distal part of the femoral stem [1]. Various techniques have been described in published scientific literature to extract fractured femoral stems. These methods include a metal-drilling procedure in the distal fragment (Wroblewski’s technique), trochanteric osteotomy followed by wedged device (Harris’s technique), and extraction by femoral trephines (Collis’s technique) [2-4]. In 1984, Collis was the first to report the use of a trephine reamer for extracting a damaged implant [5]. Subsequent investigations have confirmed that using a trephine reamer can safely assist in extracting the distal femoral stem [6]. Nevertheless, it is crucial to conduct precise measurements to prevent any potential difficulties. In this case, we utilized a trephine end femoral technique to remove a firmly implanted fractured distal femoral Corail stem from a patient without any cortical fractures.

## Case presentation

**Figure 1:**
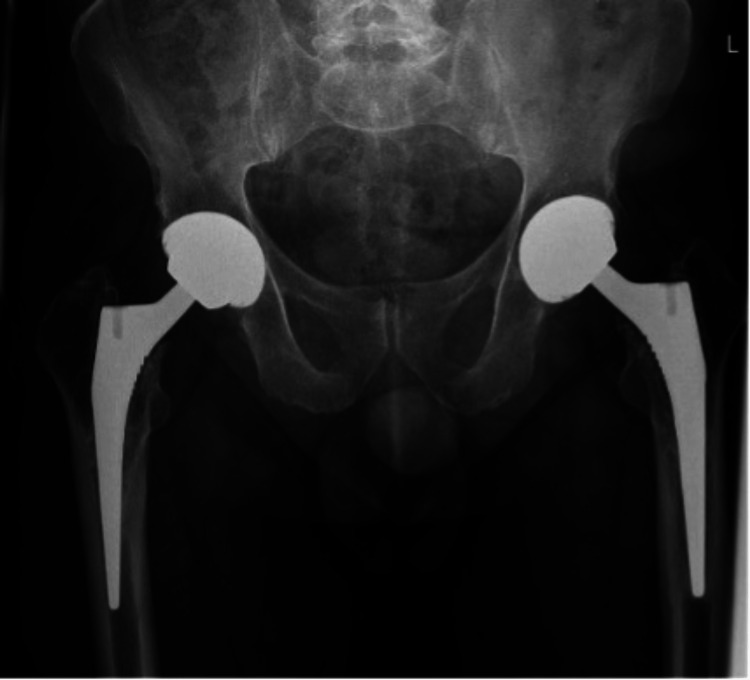
X-ray (anteroposterior view) of bilateral hip joints taken one year back showing bilateral uncemented implants in situ

**Figure 2:**
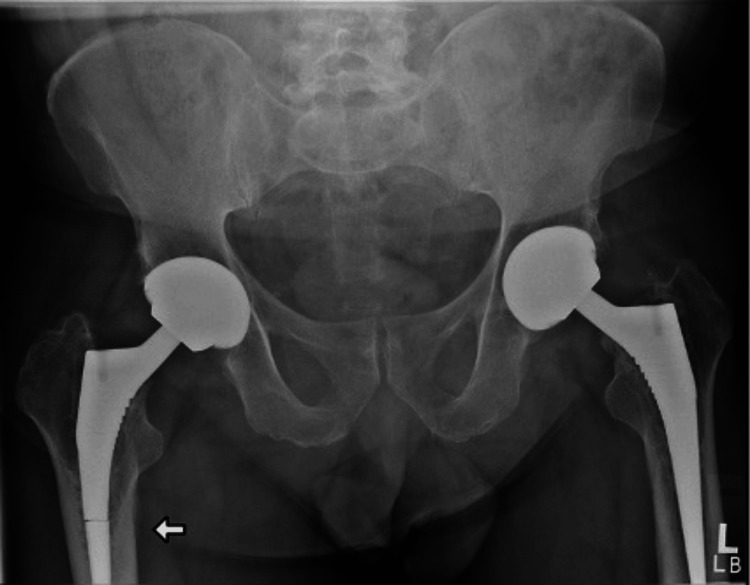
X-ray (anteroposterior view) of bilateral hip joints showing a femoral stem fracture on the right side at presentation to the clinic

**Figure 3:**
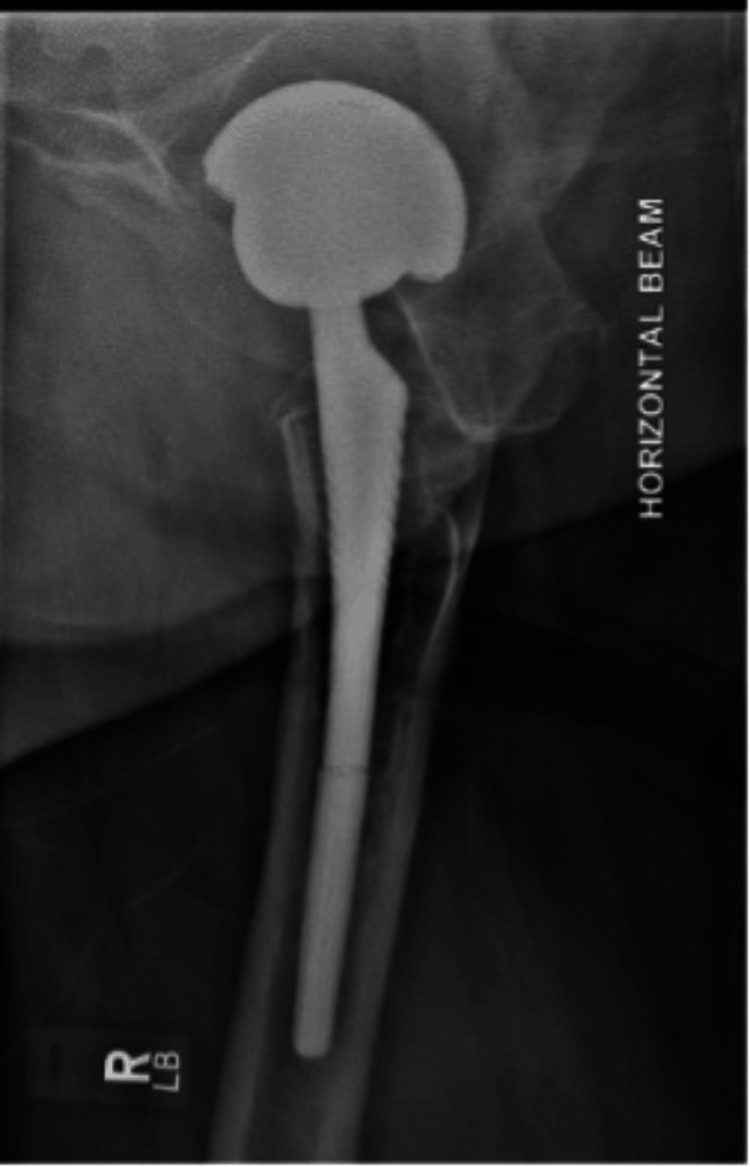
Lateral X-ray of the right hip showing the stem fracture

A 67-year-old male, with a body mass index of 35 kg/m^2^, and without any major medical comorbidities presented to the Royal Lancaster Infirmary orthopaedic outpatient department with a complaint of acute-on-chronic right anterior thigh pain. He had no other complaints and mobilised comfortably without any aids; however, the pain had gotten worse over the previous few weeks. The individual reported a history of having bilateral staged uncemented total hip replacements done around 12 years ago. The individual reported no recent history of trauma. The patient’s X-ray of the hip joints done a year back (Figure 1) and his recent X-rays (Figures 2, 3) are shown below. The plain radiological images confirmed the presence of a fracture of the Corail stem. After explaining the risks and benefits of surgery, the patient was scheduled for an elective revision hip arthroplasty.

Using the previous scar, the posterior approach was used to dislocate the hip. The proximal broken part of the stem was loose and knocked out easily. The distal broken part of the stem was removed using trephines. Following the removal of the proximal stem, the diameter of the distal part of the retrieved proximal stem was measured. We used the trephine corresponding to the diameter plus 0.5 mm. Reamers were used to lateralise the entry point, to get the right angle, and to engage the broken distal stem head-on, which was in varus alignment. Using the trephine, the broken stem was engaged and then successfully explanted. Care was taken to prevent thermal necrosis by using intermittent saline lavage while the reaming was carried out.

**Figure 4:**
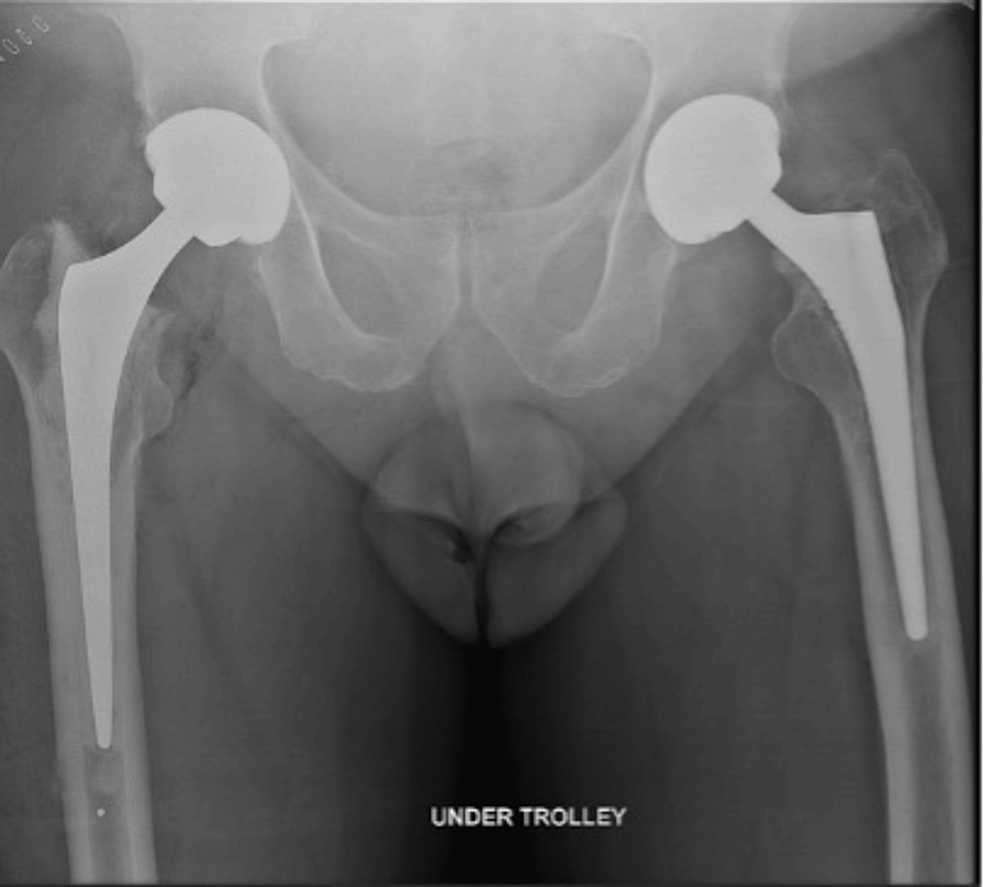
Postoperative X-ray

After the removal of the fractured femoral stem, the cup was checked and found to be well-fixed along with no significant wear in the ceramic liner. A cemented femoral revision was then performed. The patient did not suffer from any intra-operative or postoperative complications till the last follow-up (Figure 4). Initiation of ambulation full weight-bearing using crutches was commenced postoperatively. Routine deep venous thrombosis prophylaxis was given postoperatively. During the last follow-up, which occurred six weeks after the operation, the patient demonstrated the ability to ambulate without a walking aid and reported the absence of discernible discomfort in the thigh.

## Discussion

Non-traumatic femoral stem fractures are rarely reported in published scientific literature. We could find two published cases, one by Conrad et al. [7] and the other by Arafah et al. [8]. The occurrence of fractured primary and revision stems can be attributed to several reasons, including inadequate support from the surrounding bone in distally fixed stems, patients with excessive weight and BMI, increased levels of physical activity, malalignment, stem shape, and the presence of stress-risers in the stem [9]. The patient in the case report had a high BMI and a possible history of aggravated physical activity, which may have contributed to the non-traumatic stem fracture. When the diagnosis was confirmed, several methods were considered for management such as the femoral cortical window approach and the extended trochanteric osteotomy. Nevertheless, these methods are either extreme, offer restricted visibility of the distant fragment, or result in the absence of distal intra-medullary support [10]. Wroblewski developed an innovative method using specialized tools. This method involves creating a hole in the broken piece of stem and using a device coupled with a hammer to remove the broken piece from the cement layer [2]. Similarly, Harris et al. explained their method of employing a wedged device to assist in the extraction process [3]. The utilization of stainless-steel alloy in the initial hip designs greatly aided the effectiveness of these drilling procedures. Nevertheless, contemporary stems are constructed using far more durable materials, rendering these strategies less applicable. Trephine coring reamers are a well-documented method used as an additional tool to remove firmly implanted widely porous-coated and fractured cementless stems [11]. Additionally, the use of trephines can be helpful for the removal of longer, uncemented stems [9].

The trephine-based extraction technique is extremely safe and preserves cortical integrity. However, heat necrosis is a complication that needs to be kept in mind while using trephines, but intra-operative measures can help avoid this complication [5]. There may be some reservations in the mind of the orthopaedic surgeon that trephine usage may cause thermally induced necrosis. A study by Amanatullah et al. evaluated 11 fractured, uncemented, well-fixed, porous-coated femoral components involving removal using a trephine, with a minimum of two years of follow-up. The authors found that there was no compromise noted in the revision THA outcomes, with the patient’s preoperative function being restored [12]. In the above-presented case as well, after the stem fracture was corrected by revision THA, the patient had a successful postoperative course without any restrictions and regular clinical monitoring. The diagnosis had been delayed, primarily because there was no history of trauma, and possibly, the patient took the thigh pain as an age-related manifestation. This also highlights the importance of regular follow-up and counselling, which may be neglected by many patients with a THA history.

## Conclusions

Based on this case report, it can be stated that the use of trephines to remove broken femoral stems is an effective and safe method. It is important to get the correct angle so that the stem is engaged head-on. Measures such as lateralisation need to be taken to improve access to the stem if needed. Intra-operative measures help in avoiding heat necrosis while using a trephine reamer for extracting the fractured femoral stem. This case report also highlights the importance of regular follow-up and counselling, which may be neglected by many patients with a THA history, leading to a delayed diagnosis of non-traumatic femoral stem fractures. Surgical planning should include backup options such as a window technique or osteotomy.
